# Vaccine storage and cold chain monitoring in the North West region of Cameroon: a cross sectional study

**DOI:** 10.1186/s13104-015-1109-9

**Published:** 2015-04-14

**Authors:** Martin Ndinakie Yakum, Jerome Ateudjieu, Ebile Akoh Walter, Pierre Watcho

**Affiliations:** Department of Biomedical Sciences, University of Dschang, Cameroon, P.O. Box 067, Dschang, Cameroon; Divisions of Health Operations Research, Ministry of Public Health, Yaounde, Cameroon; Meilleur accès aux soins de santé (M.A.SANTE), P.O. Box 33490, Yaoundé, Cameroon

**Keywords:** Vaccine, Cold chain, Monitoring, Evaluate, EPI, Cameroon

## Abstract

**Background:**

The cold chain must be monitored continuously in order to guarantee vaccines’ quality. From field reports and previous studies, cold chain monitoring for expanded program on immunization (EPI) is still not satisfactory in Cameroon. This study was conducted to evaluate the availability and functioning of cold chain equipment as well as knowledge.

**Results:**

It was a cross-sectional study involving a multistage sampling. 3urban and 5rural districts were selected randomly from the 19 health districts of the North West region. In each district all the health facilities taking part in the EPI were targeted. Data were collected using a questionnaire administered face to face to health personnel and with an observational grid to assess availability, functioning, and monitoring of cold chain equipment and power supply. The data were analyzed using the epi-info software. A total of 70 health facilities were contacted and 65(88.6%) of them included in the study. Fifty-three (81.5%) out of 65 health facilities had at least one functional vaccine refrigerator. The national guideline of EPI was not present in 21(33.9%) health facilities. Temperature chart was complete/correctly filled in 25(50.0%) of the 50(96.2%) facilities having it. About 14 (26.9%) of the health facilities record at least one abnormal temperature during the last 2 months following data collection. Seventeen (28.3%) personnel did not know the correct vaccine storage temperature.

**Conclusion:**

The availability of vaccine storage equipment for EPI is acceptable in the North West Region of Cameroon but the capacity of those in charge to properly monitor it in all health facilities is still limited. To ensure that vaccines administered in the North West Region are stored at the recommended temperature, all District Health Services should train and regularly supervise the health personnel in charge of cold chain monitoring.

## Background

Immunization is the most precious gift that a health care worker can give a child [1] and it remains the most cost effective preventative health intervention presently known [1,2]. Vaccines are sensitive biological substances that gradually lose their potency with time [3] and this loss of potency can be accelerated when stored out of the recommended range of temperature [3,4]. Any loss of potency in a vaccine is permanent and irreversible. Consequently, a proper storage of vaccines at the recommended temperature conditions is vital so that vaccine’s potency is retained up to the moment of administration [3].

With the declaration of the reorientation of primary health care, EPI activities were incorporated into the minimum package of activities (MPAs) of all health facilities in the country. Cameroon adhered to the 2006–2015 Global Immunization Vision and Strategy (GIVS) whose one of the objectives is to ensure access to vaccines of good quality [2].

The cold chain system is a means for storing and transporting vaccines in a potent state from the manufacturer to the person being immunized [3]. It consists of a series of storage and transport links, all designed to keep vaccines within an acceptable temperature range until it reaches the users [2,4]. It makes use of human, material and financial resources as well as standards at different levels [2]. The cold chain remains a highly vulnerable point for national immunization programs in developing countries with tropical climates [5]. The success of the EPI is therefore highly sensitive to the cold chain status and hence its management should not be taken lightly.

On the other hand, primary health care providers must have adequate knowledge to manage the cold chain [6-8]. To improve management, the World Health Organization (WHO) has created a set of practice guidelines for different service levels [7]. In the same line, the ministry of public health and her partners have developed a standard operating procedure document titled “Norms and Standards for EPI in Cameroon” [2] based on the WHO guidelines adapting to the context of Cameroon.

Cold chain monitoring is still a major challenge in developing countries, including Cameroon. Previous studies suggest that only about 56% of health facilities fill their temperature charts systematically twice a day as recommended [9,10].

Cameroon persistently registers outbreaks of vaccine preventable disease such as measles even with high vaccination coverage [11]. With this situation, the quality of vaccines (being it storage problem or the efficacy) was seen as one of the major issues of the Expanded Program on Immunization in Cameroon. This study was done in the North West Region as a pilot study taking the first step in looking into the root causes of the compensation of the vaccines’ quality by evaluating cold chain monitoring. The results of this study will help to document some of the barrier to the cold chain monitoring which we can improve upon to ameliorate the immunization program thereby reducing morbidity and mortality caused by vaccine preventable diseases in Cameroon.

## Methods

It was a cross sectional study done in the North West region of Cameroon. It targeted eight randomly selected health districts of the region. All health facilities from each selected health districts were included. The North West Region of Cameroon is having an estimated total population of 1,901,579 inhabitants for the year 2013 unevenly distributed over 19 health districts and 218 health areas.

Cameroon health system is divided into 3 levels. These are the central level represented by the ministry of health; the intermediate level represented by the regional delegations of health; and the peripheral level represented by health districts. The Expanded Program on Immunization (EPI) is represented at all levels of the health system: at the central level, we have the national technical committee for EPI, at the regional level, we have the regional technical team for EPI, and the health district level we have the district health services. Vaccine procurement chain in Cameroon indicates that central store is provided by the manufacturers. This national store now supplies the regional stores which in turn supply the district stores. The district store supplies the health facilities where vaccination effectively takes place. Health facilities (HF) are categorized into integrated Health centres (Headed by a senior nurse), sub-divisional hospital headed by a medical doctor, district hospitals (first reference hospital), regional hospital (second reference hospital) and so on.

**Figure 1:**
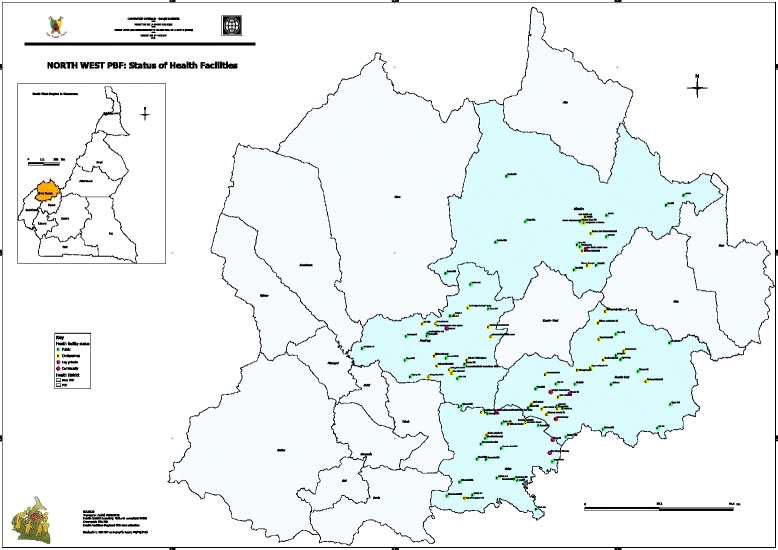
Map of Cameroon showing the regions and the health districts of the North West region.

The North West region was selected as a pilot site for the study and also due to persistence outbreaks of vaccine preventable diseases in the region. The sampling technique implemented here was multistage. First of all, the health districts were grouped as rural and urban; 5 rural and 3 urban districts were selected from their respective groups by lottery. This 5 (from 12) rural to 3 (from 7) urban selections was in conformity with distribution of the rural and urban districts among the 19 health districts. In each of the health district selected, all the health facilities taking part in the EPI were targeted for the study. One health staff from the EPI unit was taken into the study per health facility. The health staff was selected randomly from the available ones per unit. Figure 1 show the situation of North West region of the map of Cameroon and the various health district of the region.

The data collection tools were developed following the recommendation of the Norms and Standard for EPI in Cameroon. They were further pre-tested, reviewed and validated by the research team. The pre-test was done in Santa Health district of the North West region. The data were collected by one of the research team members after being trained on the consenting procedures and the data collection procedures by the other co-investigators. Data collection was by the administration of a questionnaire face to face to health staffs, direct observation of the cold chain and the consultation of the related documents to evaluate the availability and the functionality of the cold chain equipment, resources and the power supply.

The ethical approval of this study was obtained from the Cameroon National ethics committee. The informed consent form was written and since the study target health workers, each participant was given a copy to read, ask questions and free decide whether to participate or not. Acceptance was indicated by putting a signature on the informed consent form and only those who accepted and signed the form were included in the study.

The data were collected on the availability and the functionality of refrigerator, vaccine carriers, contingency plan, power supply, thermometer, guidelines, temperature chart and icepacks. The temperature chart was verified to see if it was completely filled while taking note of the number of temperature records out of the recommended intervals. The presence of foods and other substances not authorized to be put in the refrigerator as well as the arrangement of vaccines in the refrigerator was also verified and noted. On the other hand, the data collected from the health personnel were basically to evaluate their knowledge on the cold chain monitoring. These included: knowing the recommended temperature range, knowing the freeze sensitive vaccines, how to arrange vaccine in a frontly loaded refrigerator and about the open vial policy.

The data collected were coded, entered, cleaned and analyzed in the Epi Info software version number 3.5.4. In the data cleaning process, some key variables were run and the consistency and coherency of the variable checked. The major analysis done was purely descriptive running frequency and calculating the confidence interval at 95% level of confident.

## Results

### Characteristics of participants

Sixty-five from the 70 health facilities were targeted in 8 health districts included 8(12.3[5.5-22.8]%) District Health Services, 48(73.8[48.6-74.3]%) integrated health center, 5(7.7[2.5-17.0]%) sub divisional hospitals, 3(4.6[1.0-12.9]%) district hospitals and 1 (1.5[0.0-8.3]%) regional hospital. Forty-eight (73.8[61.5-84.0]%) of these health facilities were public institutions. The knowledge of 65 health service providers was evaluated of the 65 health personnel interviewed, 48(73.8[61.5-84.0]%) were female and 56(87.5[76.8-94.4]%) nurses. About 90% of the personnel had had 2 years or more working experience. Thirty-six (56.3 [31.4-56.7]%) of these health workers reported to have been trained on cold chain monitoring.

### Availability of cold chain equipment, resources and power supply in health facilities

Table 1 presents distribution of key indicators of the availability of cold chain per type of health facility.

**Table 1:** **Distribution of key indicators of the availability of cold chain per type of health facility**

**Indicator**	**Type of HF**		
	**Total (%)**	**Private (%)**	**Public (%)**
Health facility with at least one fridge in a working condition	53(81.5)	12(70.6)	41(85.4)
Number of functional fridges with a working thermometer	52(98.1)	11(91.7)	39(95.1)
Refrigerator with contingency plan pasted on the fridge	11(21.2)	4(33.3)	7(17.5)
Number of health facilities with a vaccine stock register	32(61.5)	7(41.2)	25(62.5)
Presence of the national guidelines on immunization	41(66.1)	11(64.7)	30(63.8)
Presence of electricity	53(81.5)	14(95.9)	39(84.8)

Many of the health facilities without a refrigerator needed one. The national guidelines recommend that each health facility should offer immunization activities as a fixed post. In Cameroon, all health facilities are supposed to offer a minimum package of health activities defined by the health policy and immunization is part.

### Status of cold chain monitoring in health facilities

In 26(50.0[34.8-63.4]%) of the health facilities with a temperature recording sheet, a total of 409 days were skipped without recording the temperature twice daily as recommended. Six (11.3[4.3-23.0]%) health facilities reported to have had a technical break down of refrigerator during the last 2 months and a total of 13 vials of OPV were found with the vaccine vial monitor at a discard point in 2(3.2[0.4-10.8]%) health facilities.

Concerning the 60 days preceding data collection, electricity outage was registered on the temperature recording sheet of 5(10.0[2.5-17.0]%) health facilities. A total of 239 abnormal temperatures were recorded in 14(26.9[12.3-33.5]%) health facilities. A total of 14(26.9%) vaccine fridges were exposed to overheating (temperature higher than 8°C) and 6(12%) exposed to cold (temperature lower than +2°C) in the two previous months to data collection. It is worth noting that all the vaccine refrigerators that were exposed to cold during the two months were equally exposed to overheating. Table 2 presents distribution of key indicators of the status of the cold chain stratified per type of health facility.

**Table 2:** **Distribution of key indicators of the status of the cold chain stratified per type of health facility**

**Indicator**	**Type of the health facility**		
	**Private (%)**	**Public (%)**	**Total (%)**
Temperature chart correctly filled	7(55.0)	18(47.4)	25(50.0)
Temperature at the moment of data collection found between + 2 to +8	11(95.5)	24(61.5)	35(70)
Number of HF with at least one temperature out of the +2 to +8 during the two previous month to data collection	4(36.4)	10(25.0)	14(26.4)
Icepack adequately parked	11(95.5)	31(77.5)	22(42.3)
Number of HF in which vaccines were adequately parked	7(75.0)	24(60.0)	31(59.6)
Number of HF in which diluents were adequately parked	11(95.5)	31(77.5)	42(80.8)
Number of functional fridges with a temperature recording sheet	12(100.0)	38(95.0)	50(96.2)
Number of HF in which stock register included diluents	4(46.45)	16(59.3)	20(57.1)
Number of HF in which vaccine storage was proper	7(75.0)	15(37.5)	22(42.3)

It should be noted on Table 2 that the rate of abnormal temperature recorded during the data collection period was significantly higher in public health facilities than in the private. This could be caused by the fact that many of the public health facilities did not store vaccine properly. Improper storage was mostly due to presence of food in the vaccine refrigerator, vaccines not kept at the correct compartment, inadequate air circulation, and absence of icepacks in the refrigerators.

### Knowledge of the health workers on the monitoring of the cold chain

Table 3 presents the distribution of key indicators of knowledge of health personnel on cold chain monitoring.

**Table 3:** **Distribution of the key indicators of knowledge of health personnel on cold chain monitoring**

**Indicator**	**Frequency (%)**	
	**Trained**	**Not trained**
Fridge should be turned on at least 24 hrs after its arrival	18(50.0)	18(64.3)
Vaccine should be kept in the fridge 6 to 8 hours after turning it on	28(77.8)	18(64.3)
Correctly know the open vial policy as applied to TT	22(61.1)	16(57.1)
OPV can be frozen and is not damage	24(66.7)	13(48.1)
OPV can still be used two days after the vial is opened	34(94.4)	22(81.5)
Vaccines are stored in the health facilities at a temperature between +2°C to +8°C	428(77.8)	15(62.1)
Vaccines are stored in the District Health Services at a temperature between +2°C to +8°C	22(66.4)	9(42.9)
Vaccines should be store in the HF only for a maximum of 1 month	13(41.9)	6(30.0)

It can be noted from the Table 3 that only 37.3% of the health worker knew the correct duration in which vaccines should be stored in the health facilities.

It should be note that 20 out of 53 health personnel did not know the correct place to place the measles vaccine in the refrigerator.

## Discussion

This study was conducted to evaluate availability and functioning of cold chain equipment as well as knowledge and practices of health personnel in its monitoring in the North West Region of Cameroon. About 81.5% of the health facilities participating in the Expanded Program on Immunization in the North West Region had a functional refrigerator. At the moment of data collection, temperature out of the recommended vaccine storage temperature range of +2°C to +8°C was recorded in 32.7% of health facilities that had a functional thermometer. In total, 36.9% health facilities had 239 abnormal temperatures during two previous months. Up to 43.8% did not know the recommended vaccine temperature storage range.

The fact that only some selected health facilities in the North West were included predisposes the study to selection bias. Collecting data by interview limited the result only on what the participants said. Therefore liar and loss of memory may bias the results obtained. Also, not all the targeted health facilities and health personnel accepted to participate. This non response rate may also bias the final results of the study found. The act of collecting data by observation can also induce a measurement bias. The fact that the study was done only at a particular period of the year can also bias the results since we could hardly know what happens throughout the year. Also, recording temperature twice a day or at the moment of data collection with a thermometer may not actually tell to what extent vaccines were exposed to sub-optimal temperatures. This study only reported the exposure of vaccines to sub-optimal temperature at the storage facilities and vaccines can equally be exposed to sub-optimal temperatures during transportation.

However, the selection of the health district, health facilities and the personnel that participated in this research was done randomly. Therefore this study is less likely to be explained by a selection bias. Also, the data collectors were trained before being sent to the field and they were thoroughly supervised during the collection. Therefore, the results of this study are less likely to be explained by measurement bias. It can thus be said that the result presented by this research is a true picture of what happens in the North West Region when talking about the cold chain.

This research indicates that the availability of cold chain is satisfactory. About 81.5% of the participating health facilities had at least one functional vaccine refrigerator and 98.4% of the health facilities with a functional refrigerator had at least one source of power supply. This is not very different from the result of a study published in 2013, in which only 70% of the health facilities had a functional vaccine refrigerator [10] with about 85% of the participating health facilities had at least one sources of power supply [10]. However, the slight difference observed may be due to sample variation or still, it may be because of the fact that the later study selected only districts already noted for low vaccination coverage and former selected districts randomly in one of 10 health regions of the country. Some of these health facilities had refrigerators but thermometers were still to be provided (6%). This same problem has been documented by many authors working independently [9,10,12]. The health facilities without a functional cold chain are forced to go to the nearest health facilities having cold chain in search of vaccines each time they are to organize an immunization session and to take these vaccines back to those facilities after the sessions as recommended by the national SOP [2]. This upward and downward movement with the vaccines predisposes the vaccine to overheating and long term exposure to light which can damage the vaccines and handicap the expanded program on immunization seriously [2-4]. This situation is a source of doubt about the immunization of vaccinated children. This can explain a situation observed in Cameroon in which more than half of people with measles were reported to have been vaccinated against it [11]. Vaccine wastage rate may increase and it can also lead to vaccine stock out. The work load of the health worker is increased but the main objective of the immunization which is to effectively immunize children is not attained [13-20]. Although some of the health facilities lack a functional cold chain because of lack of resources, others fail to maintain the cold chain equipment when they are bad. Buying cold chain equipment and supplying them health facilities that lack them and instituting a maintenance system may help the situation [19-21]. However, the 69.5% that had an alternative power source different from their main source were seen at the positive end. It was documented in a study in Nigeria that irregular power supply of health facilities and absence of standby generator were major risk factors of loss of vaccine potency [22].

The status of cold chain monitoring did not meet recommendation in guidelines [2]. At the moment of data collection, refrigerator’s temperature within the recommended vaccine storage temperature range of +2°C to +8°C was recorded in 67.3% of health facilities that had a functional thermometer. This is not very different from the proportion documented in Cameroon in 2013 which 70.1% [10] of the vaccine refrigerators had a temperature within the recommended range during the data collection. The situation seems to be better in Ethiopia in the year 2000 with 88.3% of the vaccine refrigerator recorded temperatures within the recommended range during the data collection [12]. The apparent higher proportion of health facilities with cold chain in recommended range in Ethiopia could be explained by the fact that the recommended temperature range in the study in Ethiopia was 0°C to +8°C [12]. In any cases, these studies were consistent in underlying that in10 to 33% of health facilities EPI vaccines administered are exposed to ranges of temperatures that question its efficiency and safety. This problem of vaccine exposure to abnormal temperatures is a worldwide issue and has been documented from many studies [9-11,13-15,21-23]. This could be explained by the low proportion of the health workers having access to national guidelines, trained on how to monitor the cold chain, supervised, and using the contingency plan, frequency power outage, [21,22].

About 11.3% of the health facilities reported to have had a refrigerator breakdown during the last 2 months. Refrigerator breakdown was also documented in a study conducted in Ethiopia in 2000 in which 31.3% of the health facilities reported to have registered refrigerator breakdown [9]. Breaking down of refrigerators induces temperature increasing and exposure of vaccine to unacceptable ranges of temperature and is expected to accelerate its loss of potency. It has to be prevented by making available and training health personnel on cold chain contingency maintenance plans.

Temperature recording was complete and up to date in 49.0% of the health facilities with a temperature recording sheet. A similar situation was documented by in Cameroon 2013as 40.7% [10] and in Ethiopia in 2000 as 57.8% [9]. The SOP in Cameroon recommends that the temperature should be registered twice daily every day including weekends and holidays [2-4]. This may be explained by lack of motivation, training and supervision of health personnel.

This study also documented the need to train health personnel in cold chain monitoring. About 71.7% of the participating health personnel were able to know the recommended temperature storage range (+2°C to +8°C). About 62.5% of the health personnel did not know the correct time interval that must be waited before starting a fridge after the arrival. Close to half of them did not know how to store vaccines in the refrigerator… This condition has been documented in Mozambique in 2007 in which 52% were able to know the recommended temperature storage range [21]. This could be explained by the absence of training and supervision [23]. Periodic assessment and response to training requirements should contribute in improving the situation. This training should be based on standardized guidelines on cold chain monitoring.

## Conclusion

The availability of vaccine storage equipment can be acceptable in the North West Region of Cameroon. If well utilized, these are enough to attend the desired level of vaccination coverage which can enable herds’ immunity (group immunity). However, the availability of the power sources and alternative sources was not very good. The number of health facilities using electricity as their primary power source predominates but the major challenge of this type of power supply is irregularity in the supply.

The cold chain monitoring status in the North West Region is inadequate to ensure proper vaccine storage. There is a call for concern on the status of the cold chain monitoring since it can cripple the immunization program. Stake holders such as the Ministry of health, Regional Delegation and the various health districts should take this as a major preoccupation.

It is true that many of the health personnel knowledge and practices on cold chain monitoring was not too bad, but a lot still needs to be done to improve on this aspect. Potential factors associated, likely to be responsible of this low level of knowledge and practices include insufficient/no training, insufficient/no supervision, absence of the SOP for vaccine storage and so on. Acting on theses, can ameliorate their practices and the status of cold chain as a consequence, which will have as an impact vaccination of children with a well potent vaccine and effective immunization rate increase and the elimination and eradication of the vaccine preventable diseases in the North West Region and the Cameroon as a whole.

### Recommendations

#### To the District and Regional health authorities

- to map access to power supply in health facilities in charge of implementing EPI activities and provide those with no access to hydroelectricity with alternative sources
- ensure that all health facilities implementing EPI activities have the Standard Operating Procedure for EPI in Cameroon.
- organize to prevent power interruption in health facilities by stocking alternative sources of power like solar, gas and kerosene,
- continuously train health personnel on cold chain monitoring
- supervise health personnel the monitoring cold chain at reasonable periodicity

## Abbreviations

BCG: Calmette-Guérin Bacillus (*vaccine*)
DPT-HepB + Hib: Diphtheria, Pertusis, Tetanus and Hepatitis B + *HaemophilusInfluenzae*type b
EPI: Expanded Programme on Immunization
HF: Health facility
GIVS: Global Immunization Vision and Strategy
MPAs: Minimum Package of Activities
OPV: Oral Polio Vaccine
SOP: Standard Operating Procedure
TT: Tetanus Toxoid
VVM: Vaccine Vial Monitor
